# Beyond Gamma: Synergistic Potential of 10 Hz Alpha and 40 Hz Gamma Photobiomodulation in Alzheimer's Disease Treatment

**DOI:** 10.1002/alz70855_105314

**Published:** 2025-12-24

**Authors:** Lew Lim, Michael A Staelens, Barbara Truglia, Davide D Lazzari, Elisabetta Di Gregorio, Karthik Shankar, Janine Liburd, Nazanin Hosseinkhah, Mahroo Karimpoor, Jack A. Tuszynski

**Affiliations:** ^1^ Vielight Inc., Toronto, ON, Canada; ^2^ University of Alberta, Edmonton, AB, Canada; ^3^ Okanagan College, Kelowna, BC, Canada; ^4^ Universita de Milano, Milan, Lombardy, Italy; ^5^ Politecnico of Turin, Turin, Piedmont, Italy; ^6^ Polytechnic University of Turin, Turin, Piedmont, Italy

## Abstract

**Background:**

Gamma (40 Hz) sensory stimulation has been proposed as a non‐pharmaceutical therapy for Alzheimer's disease (AD) due to its association with reduced pathology in animal studies. However, the focus on gamma overlooks the potential benefits of other frequencies, such as 10 Hz (alpha), which has demonstrated comparable or greater outcomes in animal models. Photobiomodulation (PBM), a non‐invasive intervention targeting specific brain regions, has shown promising results in small human studies. Combining 10 Hz and 40 Hz PBM may exploit their distinctive advantages while oncovering additional benefits. This study evaluates the effects of PBM at 10 Hz, 40 Hz, and other frequencies, synthesizing in vitro findings on tubulin dynamics with evidence from animal and human studies to refine therapeutic protocols for AD.

**Method:**

Tubulin polymerization and depolymerization were investigated *in vitro* using microscopy and Raman spectroscopy, with PBM applied at 3 Hz, 10 Hz, 40 Hz, 120 Hz and 1000 Hz. Findings were contextualized with data from animal and human studies to examine PBM effects delivered transcranially and intranasally.

**Result:**

PBM at 10 Hz induced tubulin depolymerization, potentially destabilizing misfolded proteins associated with neurofibrillary tangles and beta‐amyloid, while priming neurons for microtubular renewal. PBM at 40 Hz pivoted tubulins to polymerize, potentially stabilizing mircotubular structures and supporting neuronal network integrity. Higher frequencies such as 120 Hz and 1000 Hz, further polymerized tubulins, reinforcing microtubules and functional connectivity. Notably, 40 Hz appeared to have served as a transitional frequency, bridging the priming effects of 10 Hz and the reinforcement provided by higher frequencies. Combining 10 Hz and 40 Hz may yied superior clinical outcomes in AD and should be considered for inclusion in clinical trial protocols.

**Conclusion:**

Relying solely on 40 Hz gamma stimulation may limit therapeutic efficacy by neglecting the complementary benefits of 10 Hz. This study highlights the synergistic effects of 10 Hz alpha and 40 Hz gamma PBM, with higher frequencies potentially offering additional advantages. PBM's ability to modulate microtubular dynamics provides a foundation for exploring advanced frequency combinations. Integrating artificial intelligence could further optimize personalized, frequency‐specific PBM protocols, paving the way for more effective AD interventions.

